# *Legionella pneumophila* infection activates bystander cells differentially by bacterial and host cell vesicles

**DOI:** 10.1038/s41598-017-06443-1

**Published:** 2017-07-24

**Authors:** Anna Lena Jung, Christina Elena Herkt, Christine Schulz, Kathrin Bolte, Kerstin Seidel, Nicoletta Scheller, Alexandra Sittka-Stark, Wilhelm Bertrams, Bernd Schmeck

**Affiliations:** 1 Institute for Lung Research, German Center for Lung Research, Universities of Giessen and Marburg Lung Centre, Philipps-University Marburg, 35043 Marburg, Germany; 2 Department for Cell Biology, Philipps-University Marburg, 35043 Marburg, Germany; 3 Department of Medicine, Pulmonary and Critical Care Medicine, University Medical Center Marburg, Philipps-University, 35043 Marburg, Germany; 4 Labor Berlin Services GmbH, 13353 Berlin, Germany

## Abstract

Extracellular vesicles from eukaryotic cells and outer membrane vesicles (OMVs) released from gram-negative bacteria have been described as mediators of pathogen-host interaction and intercellular communication. *Legionella pneumophila* (*L*. *pneumophila*) is a causative agent of severe pneumonia. The differential effect of bacterial and host cell vesicles in *L*. *pneumophila* infection is unknown so far. We infected THP-1-derived or primary human macrophages with *L*. *pneumophila* and isolated supernatant vesicles by differential centrifugation. We observed an increase of exosomes in the 100 k pellet by nanoparticle tracking analysis, electron microscopy, and protein markers. This fraction additionally contained Legionella LPS, indicating also the presence of OMVs. In contrast, vesicles in the 16 k pellet, representing microparticles, decreased during infection. The 100 k vesicle fraction activated uninfected primary human alveolar epithelial cells, A549 cells, and THP-1 cells. Epithelial cell activation was reduced by exosome depletion (anti-CD63, or GW4869), or blocking of IL-1β in the supernatant. In contrast, the response of THP-1 cells to vesicles was reduced by a TLR2-neutralizing antibody, UV-inactivation of bacteria, or – partially – RNase-treatment of vesicles. Taken together, we found that during *L*. *pneumophila* infection, neighbouring epithelial cells were predominantly activated by exosomes and cytokines, whereas myeloid cells were activated by bacterial OMVs.

## Introduction

*Legionella pneumophila* (*L*. *pneumophila*), a gram-negative bacterium, is described as a causative pathogen of pneumonia^1^. Alveolar macrophages, which protect the lung from inhaled microorganisms and other insults, are their main host cell in the human lung. Infection of macrophages with *L*. *pneumophila* leads to a broad activation of signalling pathways, triggered by both extracellular receptors, such as TLRs, and intracellular receptor molecules, such as RIG-I and NAIP5^2^. The initial proinflammatory activation pattern that can be observed in macrophages upon infection is defined by the up-regulation of TNF-α, IL-6 and IL-1β^3^. *L*. *pneumophila* are phagocytosed and contained in vesicular cytosolic bodies, the phagosomes, that are bound for lysosomal degradation. Legionella actively blocks this mechanism of cellular defence by transmembrane secretion of effector proteins into the host cell via the dot/Icm type IV secretion system^4^. These factors are instrumental to formation of a replication niche, the *Legionella*-containing vacuole (LCV), which is recruited from ER-derived vesicles. In this way, the LCV gains structural resemblance to the ER, which is hypothesized to protect it from the endocytic pathway^5^. In the LCV, Legionella replicates for about 14 h until host cell lysis^6^. Of note, the secreted effectors are also recognized by cytosolic sensors such as NAIP5 and NLRC4, leading to further activation of the host cell by caspase-I cleavage^7^. While limiting intracellular *Legionella* growth^8^, the activation of the host cell by intracellular pathogens also leads to the release of stimulatory extracellular vesicles (EVs)^9^.

Eukaryotic cells can produce various kinds of EVs, which are classified by their sub-cellular origin. Apoptotic cells secrete larger apoptotic bodies, EVs shedding from the plasma membrane are named microparticles, and endosomal compartment-derived EVs are defined as exosomes. EVs are composed of a lipid bilayer, they are carrying transmembrane proteins, and they are transporting intraluminal cargo (RNA and proteins) to recipient cells^10^. Exosomes and microparticles can be distinguished by certain marker proteins, such as tetraspanins (e.g. CD9, CD63, CD81), Alix and Tsg101, which are present on exosomes due to their endosomal origin. EVs are found in various body fluids and are an important means of communication between eukaryotic cells^11^. Their uptake by recipient cells can be induced via receptor-ligand interaction, endocytosis, phagocytosis or membrane fusion to release the cargo^12^. The release of EVs depends on the physiological state of the donor cell such as the condition of the microenvironment^13, 14^. Under inflammatory conditions, the secretion of EVs can increase, as shown for sarcoidosis, asthma or *Mycobacterium bovis* infection^9, 15, 16^.

The secretion of EVs seems to be conserved throughout evolution, as not only higher eukaryotes are producing EVs, but also prokaryotes^17^. Gram-negative bacteria form spheroid, nano-sized EVs, the so called outer membrane vesicles (OMV)^18^. These allow the transport of diverse virulence factors (e.g. toxins and enzymes) as well as lipopolysaccharides (LPS), which are present on the OMV surface^19^. *L*. *pneumophila* OMVs are known to be pro-inflammatory activators of epithelial cells and macrophages^20, 21^. Additionally, they can modulate the course of infection *in vitro*. We recently published that *Legionella pneumophila*-derived OMVs promote bacterial replication in human and murine macrophages^22^.

We sought to better understand the impact of intercellular communication via EVs during the course of *L*. *pneumophila* infection and to distinguish the role of different EV subsets, namely OMVs and exosomes, on alveolar recipient cells.

## Results

### Infected cells release a heterogeneous EV population of exosomes and OMVs

**Figure 1:**
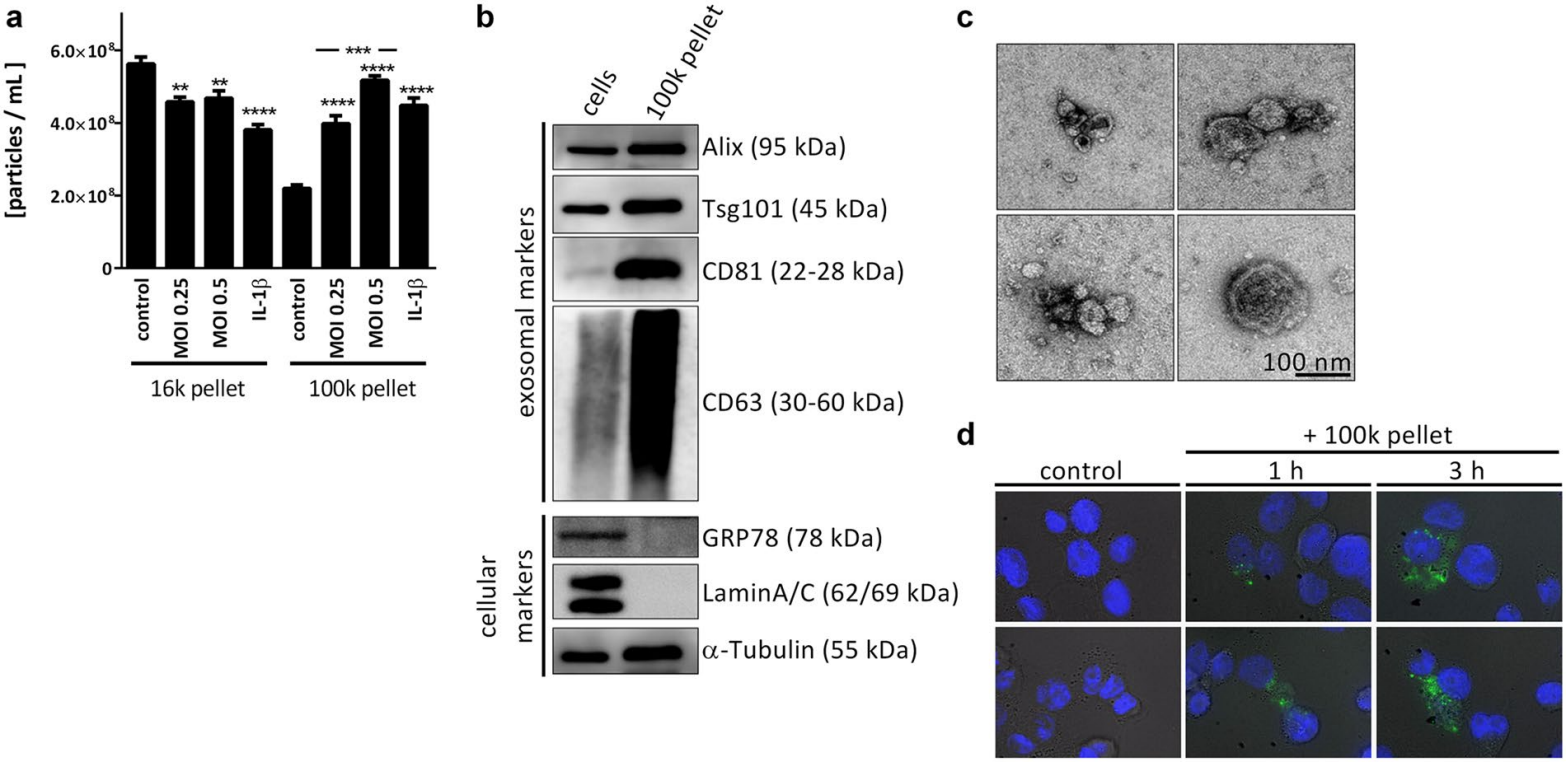
*L*. *pneumophila* infection increases the secretion of EVs in THP-1 cells. (**a**) Amount of EVs in response to *L*. *pneumophila* infection. THP-1 cells were treated with IL-1β (1 ng/mL) or infected with *L*. *pneumophila* (MOI 0.25 or 0.5, respectively) for 24 h. NTA was performed with the distinct particle fractions separated by differential centrifugation. (**b**) Western blot for exosomal marker proteins. Whole cell lysate or 100 k pellet derived from uninfected THP-1 cells were used. Equal protein amounts were loaded. (**c**) Transmission electron microscopy with 100 k pellet. Purified EVs from THP-1 cells were fixed and visualized after negative staining with uranyl acetate. Scale bar represents 100 nm. (**d**) Uptake of A549-derived EVs by THP-1 cells. A549 cells were stained with the membrane dye PKH67. EVs were collected (100 k pellet) and incubated with THP-1 cells for 1 or 3 h, respectively. Nuclei were stained by DAPI and pictures were taken with an original magnification of 630x. A: Data are shown as mean + SEM of three independent experiments. **p < 0.01, ***p < 0.001, ****p < 0.0001. B–D: representative results.

THP-1 cells respond to *L*. *pneumophila* infection with increased secretion of pro-inflammatory cytokines (TNF-α, IL-1β, IL-6 and MCP-1, Supplementary Fig. 1) and notably EVs (Fig. 1a). These nano-sized vesicles were collected by a 100,000 xg ultracentrifugation step and then quantified by nanoparticle tracking analysis (NTA). The amount of particles/mL in this so called 100 k pellet increased with multiplicity of infection, which could also be observed upon administration of the sterile stimulant IL-1β. Similarly, primary human blood-derived macrophages responded to an infection with *L*. *pneumophila* with an increased secretion of EVs (Supplementary Fig. 2b). In contrast, the amount of the larger microparticles in the 16k pellet was found to be slightly reduced after infection (Fig. 1a). Within the 100 K EV fraction, we detected an increase of the exosomal markers Alix and Tsg101 after *L*. *pneumophila* infection Fig. 1b) which we could also show after IL-1β treatment (Supplementary Fig. 2a). We could further characterize this fraction by the presence of the tetraspanins CD63 and CD81, which are exosomal marker proteins, and the absence of cellular markers GRP78 and LaminA/C (Fig. 1b). EVs from the 100 k pellet were visualized by transmission electron microscopy, showing their spherical shapes of approximately 20–100 nm in diameter (Fig. 1c). The presence of *Legionella*-LPS in the 100 k pellet (Supplementary Fig. 2c) indicates that the 100 k pellet released from infected THP-1 cells consists of a mixed population of exosomes and microparticles (both secreted from eukaryotic cells), and of bacterial OMVs carrying LPS. The amount of bacterial OMVs in the 100 k pellet of *L*. *pneumophila* infected THP-1 cells was determined by relative LPS quantification and equalled 0.5 µg OMVs. As our approach lays a focus on the stimulatory potential of the EV fraction on uninfected recipient cells, we visualized the uptake of PKH67-stained EVs from A549 cells by THP-1 cells by immunofluorescence analysis (Fig. 1d).

### Recipient A549 cells and THP-1 cells respond differently to stimulation with the 100 k pellet or vesicle-free cytokine-containing supernatant of infected and control THP-1 donor cells

**Figure 2:**
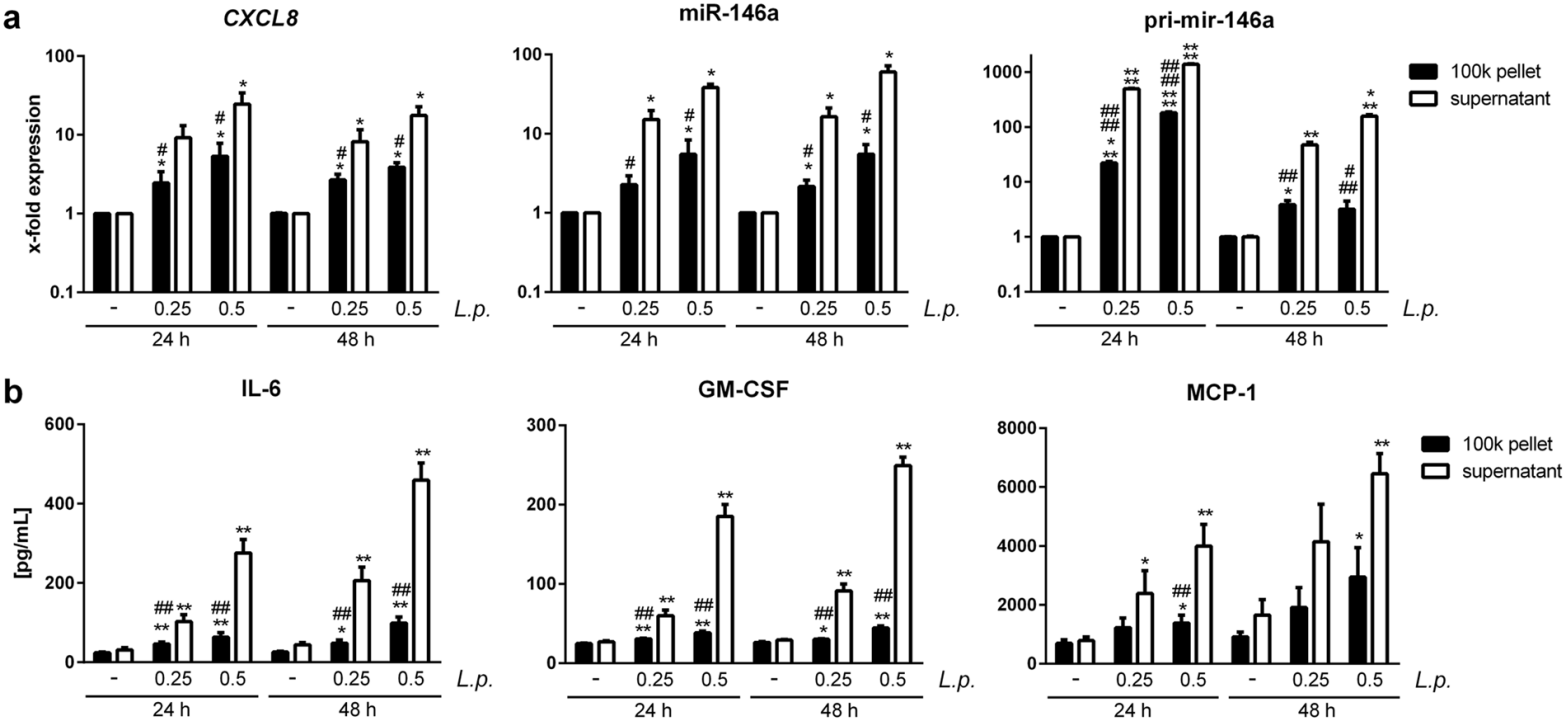
*L*. *pneumophila* induced cytokines elicit a pro-inflammatory response in non-infected A549 cells. (**a**,**b**) Response of A549 cells to vesicle-free supernatant or 100 k pellet from *L*. *pneumophila*-infected THP-1 cells. A549 cells were stimulated with vesicle-free supernatant or 100 k pellet from THP-1 cells infected with *L*. *pneumophila* (*L*.*p*.) at an MOI of 0.25 and 0.5, respectively, for 24 h. A549 cells were incubated as indicated for 24 or 48 h, respectively. RNA and supernatant were collected. (**a**) qPCR was performed for expression of *CXCL8*, miR-146a and pri-mir-146a. (**b**) Magnetic multiplex ELISA was performed. Results for IL-6, GM-CSF and MCP-1 are shown. Data are shown as mean + SEM of at least three independent experiments. *p < 0.05, **p < 0.01. *Compared to corresponding control, ^#^compared to corresponding supernatant treated sample.

**Figure 3:**
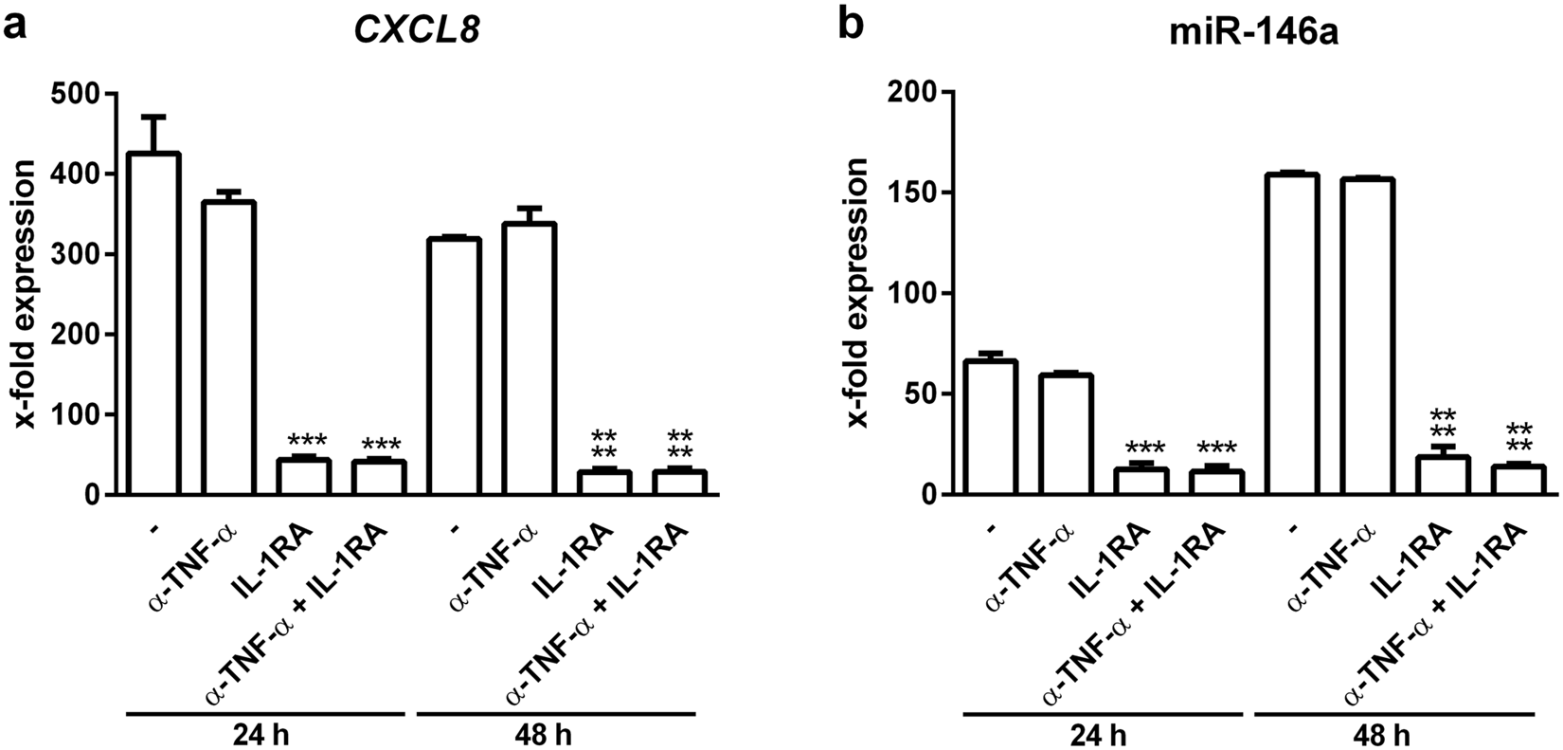
THP-1-derived IL-1β induces pro-inflammatory response in A549 cells. (**a**,**b**) Response of A549 cells to vesicle-free supernatant from *L*. *pneumophila*-infected THP-1 cells. THP-1 cells were infected with *L*. *pneumophila* (MOI 0.5) for 24 h. A549 cells were pre-incubated for 1 h with a neutralizing TNF-α antibody (α-TNF-α) or an IL-1 receptor antagonist (IL-1RA) alone or in combination, respectively. Cells were then incubated with the vesicle-free supernatant for 24 or 48 h, respectively. RNA was collected and used for expression analysis of *CXCL8* (**a**) and miR-146a (**b**). Data are shown as mean + SEM of three independent experiments. ***p < 0.001, ****p < 0.0001 compared to corresponding supernatant treated cells.

**Figure 4:**
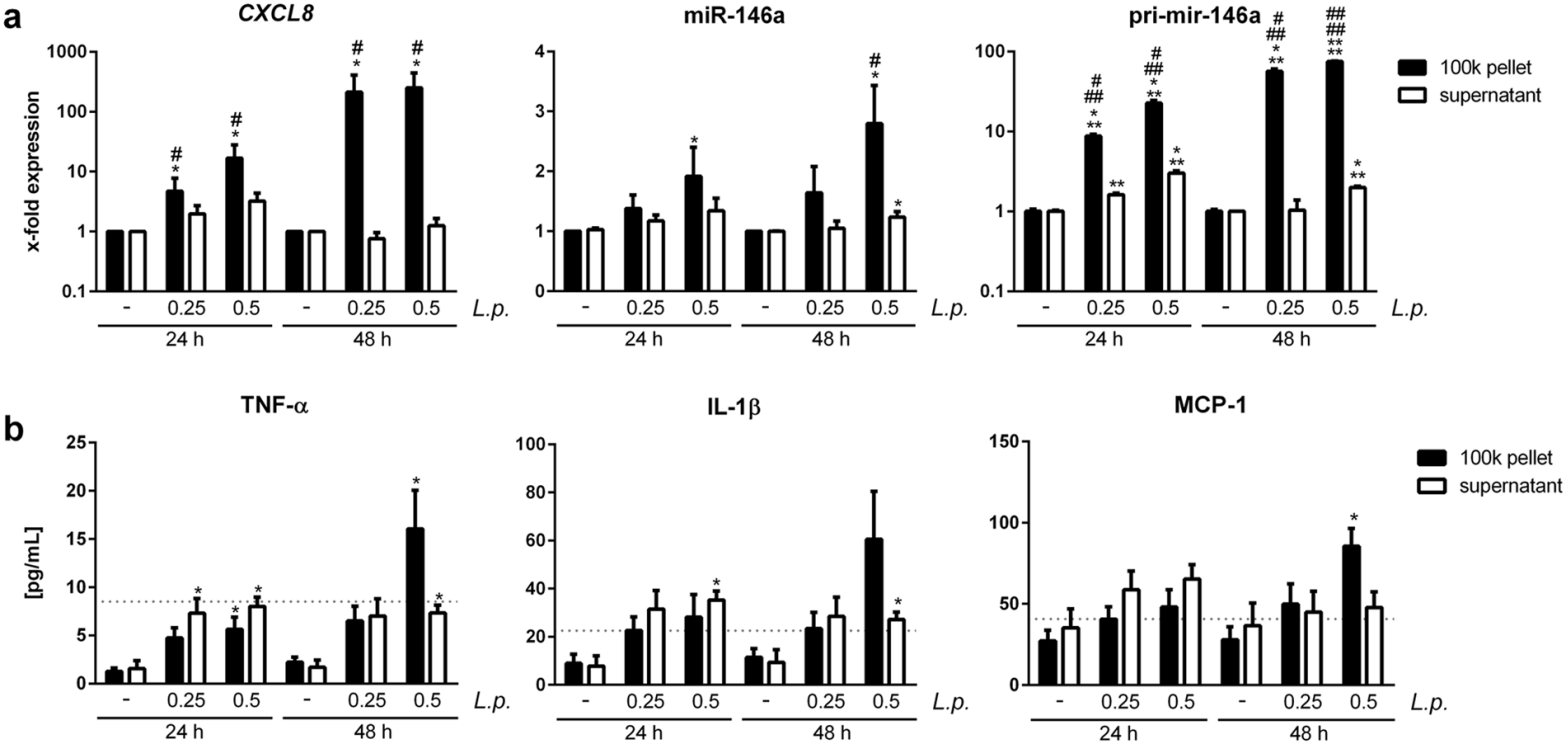
*L*. *pneumophila* induced EVs elicit a pro-inflammatory response in non-infected THP-1 cells. (**a**,**b**) Response of THP-1 cells to vesicle-free supernatant or 100 k pellet from *L*. *pneumophila*-infected THP-1 cells. THP-1 cells were stimulated with vesicle-free supernatant or 100 k pellet from THP-1 cells infected with *L*. *pneumophila* (MOI 0.25 and 0.5, respectively) for 24 h. Recipient THP-1 cells were incubated for 24 or 48 h, respectively. (**a**) qPCR was performed for expression of *CXCL8*, miR-146a and pri-mir-146a. (**b**) Magnetic multiplex ELISA was performed. Results for TNF-α, IL-1β and MCP-1 are shown. Dashed lines indicate the input of the cytokine contained in the vesicle-free supernatant of infected THP-1 cells (MOI 0.5) before adding it to the recipient cells. Data are shown as mean + SEM of at least three independent experiments. *p < 0.05. *Compared to corresponding control, # compared to corresponding supernatant treated sample.

The stimulatory potential of the 100 K pellet on recipient cells needs to be carefully evaluated with respect to the variety of immune-stimulatory compounds that the pellet contains. To this end, the stimulatory capacity of EVs and cytokines that are both released from *L*. *pneumophila*-infected THP-1 cells was tested on different recipient cells. A549 cells responded with increased expression of *CXCL8* and the anti-inflammatory microRNA-146a (miR-146a) to the cytokine-containing vesicle-free supernatant from infected THP-1 cells (Fig. 2a). Additionally, the secretion of IL-6, GM-CSF and MCP-1 was elevated in supernatant-treated A549 cells (Fig. 2b). In contrast, their response to stimulation with a 100 k pellet derived from infected THP-1 cells was significantly weaker, as assessed by the same parameters (Fig. 2a,b). This was correspondingly observed for primary human alveolar epithelial cells (Supplementary Fig. 3a,b). To establish a more profound transcriptional profile, we also measured the expression of *SOD2*, *CXCL1*, *IFN-β*, *IL-1β* and *TNFAIP2* in A549 cells treated with supernatant or with the 100 K pellet (Supplementary Fig. 6), yielding the same pattern as observed before. As miRNAs can functionally be transferred via EVs^23^ and specifically miR-146a has been demonstrated to be transported between immune cells^24^, we measured the expression of the primary transcript of miR-146a (pri-mir-146a) in recipient cells. We observed a strong induction of pri-mir-146a in A549 cells after supernatant and EV treatment (Fig. 2a). As A549 cells responded to a stronger extend to the cytokine-containing supernatant of *L*. *pneumophila*-infected THP-1 cells, we aimed to discover the responsible cytokine. The response of A549 cells to the THP-1-derived supernatant could be blocked by pre-incubation of the recipient cells with an IL-1 receptor antagonist (IL-1RA), as illustrated by the significantly reduced expression of *CXCL8* and miR-146a, while a TNFα-neutralizing antibody (α-TNF-α) had no effect on the expression of *CXCL8* or miR-146a (Fig. 3a,b). Interestingly, when THP-1 cells were used as recipient cells, they responded significantly more to the EVs than to the supernatant when assessing the same parameters (Fig. 4a,b and Supplementary Fig. 7). We performed Ingenuity Pathway Analysis (Qiagen) on the identified markers and observed significant enrichment of relevant immune pathways and events in infection, such as attraction of leukocytes, apoptosis of leukocytes and organization of cytosceleton (Supplemental Fig. 8), indicating broad activation of the recipient cells. Thus, we tried to establish the response pattern of A549 and THP-1 cells to the EV fraction.

### While THP-1 cells respond to the bacterial fraction of the 100 k pellet, A549 cells respond to the exosome fraction

**Figure 5:**
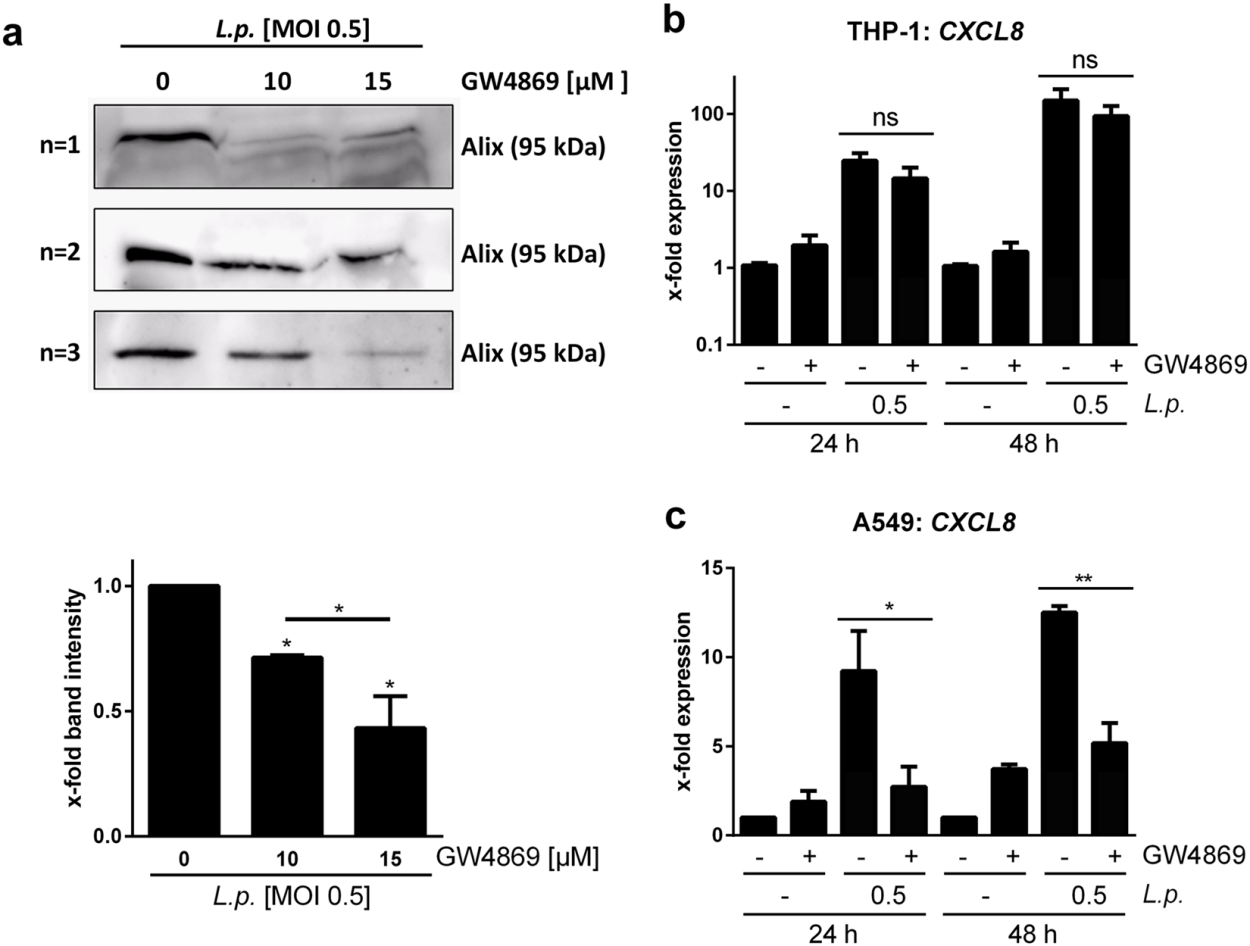
Block of exosome release does not reduce the THP-1 response to the 100 k pellet. (**a**) THP-1 cells were incubated with GW4869 (10 or 15 µM) or DMSO as a solvent control for 1 h before infection with *L*. *pneumophila* (MOI 0.5) for 24 h. A 100 k pellet was generated and Alix expression was analysed by western blot. Densitometric quantification of western blot bands is shown. Exosome secretion is reduced after GW4869 treatment. (**b**,**c**) Donor THP-1 cells were pre-incubated with GW4869 (15 µM) or the solvent control for 1 h before infection with *L*. *pneumophila* for 24 h. EVs were purified by differential centrifugation and added to THP-1 (**b**) or A549 (**c**) cells. *CXCL8* expression was analysed by qPCR after 24 or 48 h of incubation. Data are shown as mean + SEM of three independent experiments. *p < 0.05, **p < 0.01, ns: not significant.

As the 100 K pellet is heterogeneous, its stimulatory effect on recipient THP-1 or A549 cells was addressed. Therefore, specific subfractions were targeted for deletion. A reduction of exosome secretion can be achieved by GW4869, a neutral sphingomyelinase (nSMase) inhibitor^25, 26^. GW4869 was applied to the donor THP-1 cells and reduced exosome secretion was validated by western blot for the exosomal protein Alix (Fig. 5a). It is noteworthy that *L*. *pneumophila* replication or OMV secretion was not affected by GW4869 treatment of the infected THP-1 cells (Supplementary Fig. 4a,b). Recipient THP-1 cells responded with *CXCL8* mRNA expression to the exosome-depleted 100 k pellet (Fig. 5b). Conversely, recipient A549 cells showed a significant reduction of *CXCL8* mRNA expression upon incubation with the 100 k pellet from GW4869 treated THP-1 cells (Fig. 5c).

### Stimulation of A549 and THP-1 cells with enriched 100 k pellet subfractions reveals differential response patterns

**Figure 6:**
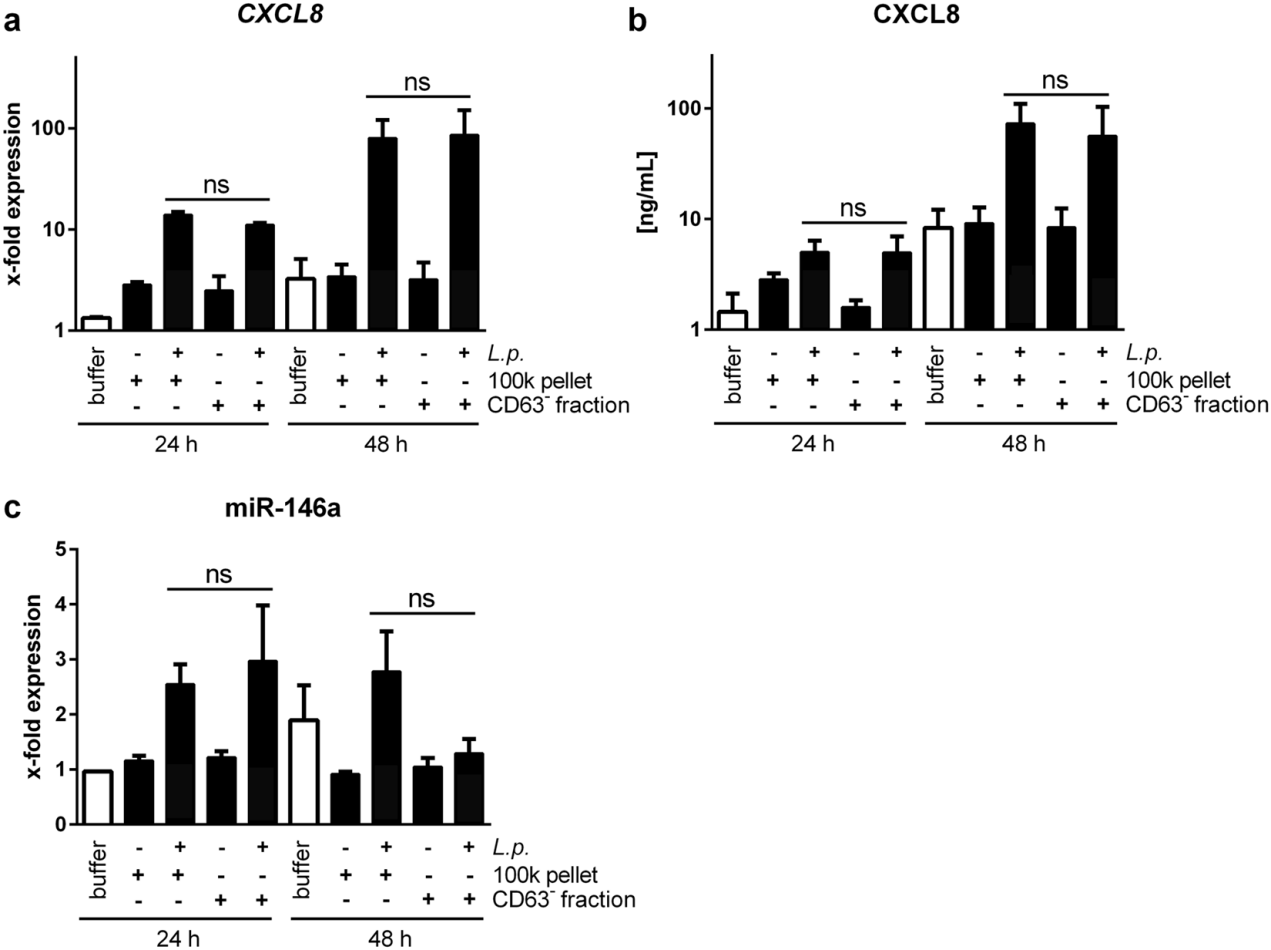
THP-1 cells respond to the CD63-negative EV fraction. (**a**,**c**) Pro-inflammatory THP-1 response depends on OMVs. 100 k pellets from *L*. *pneumophila*-infected or control THP-1 cells were generated. CD63-positive exosomes were removed by immunoprecipitation. Entire 100 k pellet or unbound EVs (CD63^−^ fraction) were used for stimulation of THP-1 cells for 24 and 48 h. Expression of *CXCL8* (**a**), CXCL8 secretion (**b**) and expression of miR-146a (**c**) were analysed. Data are shown as mean + SEM of three independent experiments. ns: not significant.

In order to pinpoint the described effects of the EVs more specifically to the bacterial or human component of the 100 k pellet, the CD63-positive exosome fraction was depleted by anti-CD63 immunoprecipitation. *L*. *pneumophila* OMVs were still present in the unbound fraction after CD63 immunoprecipitation, and naturally in the complete 100 k pellet, but not in the CD63-postitive exosome fraction, as shown by LPS dot blot (Supplementary Fig. 4c). Recipient THP-1 cells responded to the exosome-depleted fraction (CD63^−^) in the same manner as they did to the entire 100 k pellet, as illustrated on the level of *CXCL8* mRNA expression and CXCL8 release (Fig. 6a,b), pointing towards the stimulatory effect of OMVs on macrophages.

As *L*. *pneumophila* OMVs are known to activate macrophages via TLR2^22^, we analysed the involvement of TLR-ligands in the 100 k pellet of *Legionella*-infected THP-1 cells on macrophage activation by applying the EVs to mouse bone marrow derived macrophages (mBMDM) from different genetic backgrounds. TLR2/4^−/−^ macrophages did not respond with CXCL1 secretion to the 100 k pellet as wild type mBMDM did. While TLR3/7/9^−/−^ cells showed a slightly stronger secretion of CXCL1 when compared to TLR2/4^−/−^ cells, there was a substantial reduction when compared to wild type macrophage (Supplementary Fig. 5a). Irrespective of the genetic background, all three responded comparably to the stimulation with murine TNF-α (Supplementary Fig. 5b).

### THP-1 cells respond to TLR2 ligands from viable bacteria

**Figure 7:**
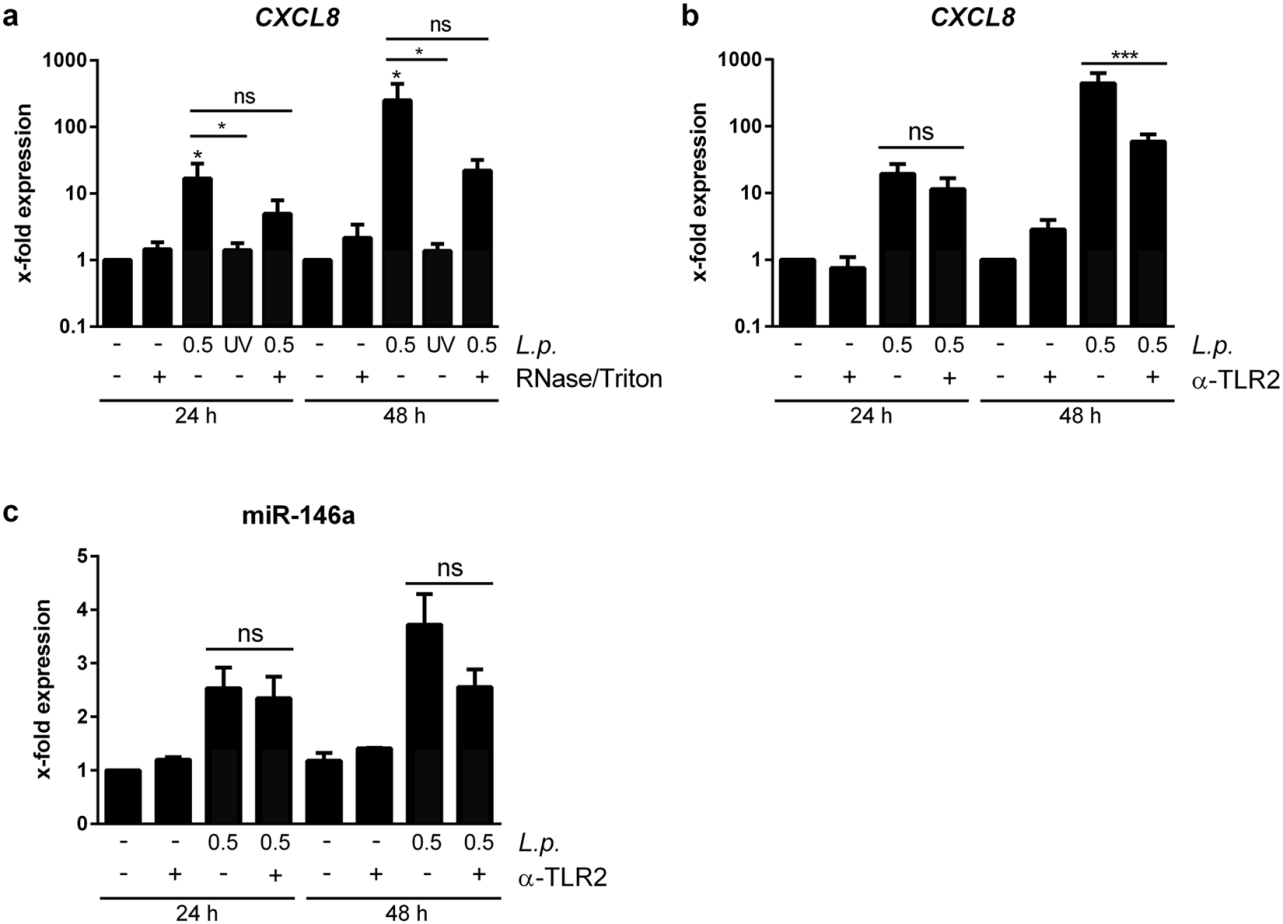
Viable *L*. *pneumophila* are critical for response of THP-1 cells to EVs. (**a**) Response of THP-1 cells to EVs from *L*. *pneumophila*-infected THP-1 cells. THP-1 cells were infected with *L*. *pneumophila* or stimulated with UV-inactivated *L*. *pneumophila* (both MOI 0.5 for 24 h). 100 k pellets were generated and left untreated or digested with RNase A (0.2 µg/µL) in the presence of 0.3% Triton X-100 for 1 h at 37 °C. The prepared EVs were added to THP-1 cells for 24 or 48 h, respectively, exposing the cells to a subcritical final Triton X-100 concentration of 0.003%. Expression of *CXCL8* was determined. (**b**,**c**) TLR2 dependent response of THP-1 cells to EVs from *L*. *pneumophila*-infected THP-1. THP-1 cells were infected with *L*. *pneumophila* (MOI 0.5 for 24 h) and the 100 k pellet was generated. Recipient THP-1 cells were pre-incubated for 90 min with a TLR2 blocking antibody (α-TLR2,+) or a control antibody (−; both 20 µg/mL). The EVs were added for 24 or 48 h, respectively. Expression of *CXCL8* (**b**) and miR-146a (**c**) was determined. Data are shown as mean + SEM of three independent experiments. *p < 0.05, ns: not significant.

In order to analyse the active bacterial compounds in the 100 k pellet to which THP-1 cells respond, the donor THP-1 cells were either treated with UV-inactivated bacteria, to date the only way to block OMV release, or the 100 k pellet was treated with Triton X-100 in combination with RNase A before administration to recipient THP-1 cells. While viable bacteria were crucial for the activating properties of the 100 k pellet on THP-1 cells, the degradation of the pellet’s RNA content yielded inconclusive results, as shown by *CXCL8* mRNA expression (Fig. 7a). Pre-treatment of the recipient THP-1 cells with a TLR2-blocking antibody also reduced the activation potential of the pellet as shown by a sharp drop in *CXCL8* mRNA expression to 13.4% (Fig. 7b). Furthermore, blockage of TLR2 diminished miR-146a expression in the recipient cells (Fig. 7c). We could thus confirm the presence of bacterial components in the pellet that require infection of donor THP-1 cells with viable bacteria and activate recipient macrophages via TLR2.

## Discussion

In this study, we have found that *L*. *pneumophila* infection of human macrophages induced the release of EVs and cytokines, which can activate uninfected bystander cells. Upon infection, we observed an increased release of smaller EVs (100 k pellet), while the larger microparticles (16 k pellet) were reduced upon infection with *L*. *pneumophila*. The 100 k pellet mainly contained exosomes, as shown by the enrichment of exosomal marker proteins on western blot and the observed size in electron microscopy (<150 nm). The increase in EV release is in line with the current literature on inflammatory conditions, as mycobacterial infection^9^, asthma^16^, sarcoidosis^15^ or acute lung injury^27^ also lead to an enhanced EV release. We further analysed which of the different uninfected recipient cells in the alveolus, namely alveolar epithelial cells or macrophages, respond to the released cytokines and the vesicle fraction upon *L*. *pneumophila* infection of neighbouring cells. We could demonstrate that alveolar epithelial cells mainly responded to the cytokine fraction by secretion of IL-6, CXCL8, GM-CSF and MCP-1 and to a weaker extend to the 100 k pellet. We hypothesized that IL-1β was the main stimulating cytokine, as it is known that macrophage derived IL-1β can prime alveolar epithelial cells, which is crucial for the recruitment of neutrophils in a murine pneumonia model^28^. To address this, blocking experiments with an IL-1 receptor antagonist were carried out, which confirmed that IL-1β was the main stimulating factor of the vesicle-free supernatant for alveolar epithelial cells. In contrast, resting macrophages responded with release of pro-inflammatory cytokines to EVs from infected donor cells, which was also observed for the infection with other intracellular pathogens elsewhere^9^. The release of CXCL8 can lead to recruitment of neutrophils, which are instrumental in clearing the infection^29, 30^. We furthermore measured the expression of additional inflammatory markers, such as *SOD2*, *CXCL1*, *IFN-β*, *IL-1β* and *TNFAIP2*. The marker pattern we observe indicates a broad pro-inflammatory activation pattern of the recipient cells.

To determine the EV component inducing the *CXCL8* expression in recipient cells, the release of exosomes was blocked by administration of GW4869, a nSMase inhibitor^25, 26^. Application of GW4869 to donor THP-1 cells before infection reduced the exosome secretion, while it did not affect *L*. *pneumophila* replication. The obtained 100 k pellet, devoid of exosomes, was equally stimulatory to recipient THP-1 cells, while it was less stimulatory to A549 cells. As alveolar epithelial cells strongly respond to IL-1β and as exosomes can transport IL-1β^31–33^, the reduction of exosome release might in turn also reduce IL-1β in the 100 k pellet, which could explain the diminished A549 response after GW4869 application to the EV-producing macrophages. The observation that macrophages still responded to the 100 k pellet might be explained by the presence of OMVs, which are released by *L*. *pneumophila* during the intracellular growth in the macrophages^20^. This is in line with our detection of *L*. *pneumophila* LPS in the EV pellet, which was not affected by GW4869 treatment. The presence of bacterial LPS in the EV fraction was also observed for *Salmonella typhimurium* infection^9^, which also secrete OMVs^34^. In another study, the EV fraction was found to contain mycobacterial lipids which could be transferred to uninfected bystander cells^35^ and could induce a pro-inflammatory response^36^. Besides lipids and LPS, bacterial proteins^37^ or even viral RNA as demonstrated for Epstein-Barr virus^38^ or Hepatitis C virus^39^ infection have been detected in EVs. To further investigate the involvement of the different EV subsets present upon *Legionella* infection, CD63-positive exosomes were depleted from the 100 K pellet derived from infected THP-1 cells. The remaining unbound EVs were still stimulatory to recipient macrophages, which is in line with the results obtained by sphingomyelinase inhibition, as both treatments lead to an exosome-depleted 100 K pellet. As there is to date no *Legionella* mutant incapable of releasing OMVs, we performed infection experiments of donor THP-1 cells with UV-inactivated bacteria and used the obtained 100 k pellet for stimulation experiments. This reduced the *CXCL8* expression in THP-1 cells in response to the EVs to baseline, suggesting that *L*. *pneumophila* OMVs are the main stimulating factor in the 100 k pellet for THP-1 macrophages. As previously published, *L*. *pneumophila* OMVs are strong pro-inflammatory stimulators for human und murine macrophages^22, 40^, which can explain the herein described results of the 100 k pellet on THP-1 cells. It has previously been published for mycobacterial infections that different EV populations have distinct stimulatory properties^41^. In this study, exosomes were not stimulatory on recipient THP-1 cells, while OMVs were. The involvement of bacterial LPS in the EV-mediated activation of THP-1 cells was shown by pre-incubation of recipient cells with a TLR2 blocking antibody, as *Legionella* LPS is recognized mainly via this membrane receptor^42^ and as the released OMVs also activate TLR2^22, 40^. Blocking of TLR2 resulted in a reduced expression of *CXCL8* in THP-1 macrophages. In accordance with this, mBMDM from TLR2/4 double knockout mice did not respond to the 100 k pellet. The involvement of TLR signalling in the pro-inflammatory response to EVs was equally observed for mycobacteria^9, 43^.

It is well described that EVs can transport different RNA species from one cell to another^23^. To address this, we tested the impact of the transported RNA cargo on *CXCL8* induction in recipient macrophages with RNase A digestion experiments. As we only observed a partial reduction in *CXCL8* response, we concluded that RNA is only an accessory stimulating factor in our system. Additionally, the induction of the anti-inflammatory miR-146a after EV stimulation of macrophages was due to a transcriptional induction and further processing of the primary transcript, as demonstrated by the increased expression of pri-mir-146a in the recipient cell. Furthermore, it has recently been demonstrated that OMVs can transport bacterial RNA^44–46^. Since EVs released during infection can contain pathogenic RNA^38, 39^, the involvement of endosomal TLRs in macrophage activation was assessed by stimulating mBMDM from TLR3/7/9 knockout animals. While mBMDM from wild type animals showed an increased secretion of CXCL1, the murine functional homologue to the human CXCL8, macrophages from mice lacking the endosomal TLRs 3, 7 and 9 were only slightly more responsive to the 100 k pellet than TLR2/4^−/−^ cells. While mechanisms of EV susceptibility in mice and men might differ, this result suggests a similar contribution of TLR2/4 and TLR3/7/9 agonists in the activation of murine macrophages by the 100 k pellet.

**Figure 8:**
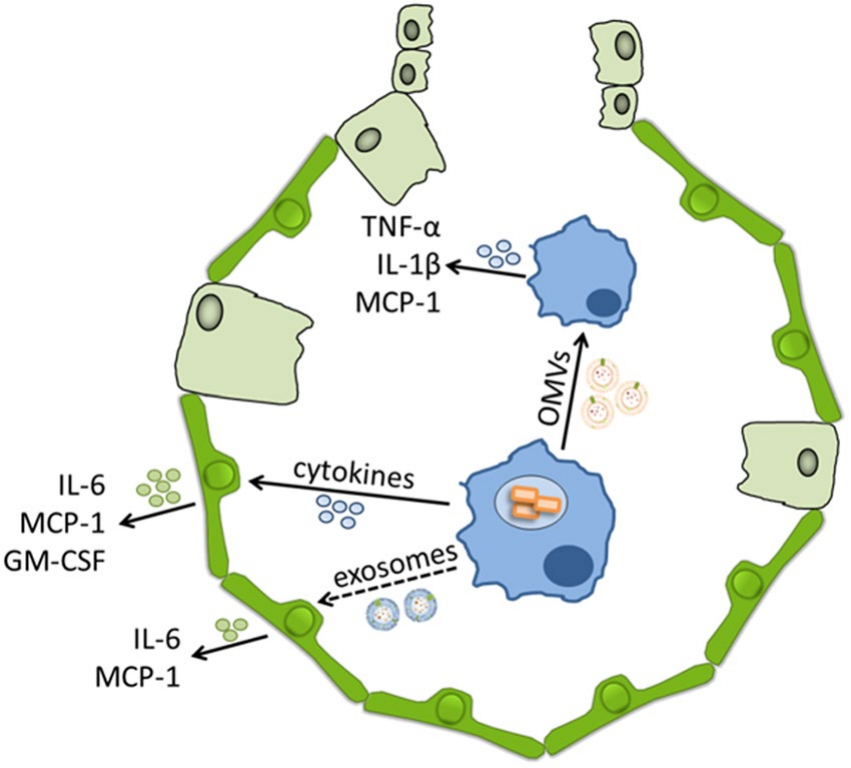
Differential microvesicles signalling in *L*. *pneumophila* infection of macrophages. The infection of macrophage-like THP-1 cells with *L*. *pneumophila* induces the release of pro-inflammatory cytokines and EVs, eukaryotic exosomes and prokaryotic OMVs. While alveolar epithelial cells mostly respond to the cytokines with a pro-inflammatory response and to a weaker extent to the exosomes, macrophages respond to the released EVs. Here, the bacterial OMVs carrying the LPS are the main stimulating agent.

We conclude that the infection of macrophages with *L*. *pneumophila* increases the release of exosomes along with the secretion of bacterial OMVs (Fig. 8). While OMVs are activating resting macrophages, leading to the secretion of pro-inflammatory cytokines, exosomes are stimulating alveolar epithelial cells. Macrophage-derived cytokines are additionally activating epithelial cells, which further enhances the amount of pro-inflammatory cytokines, potentially leading to the recruitment of alveolar macrophages and neutrophils *in vivo*. The observation that the recruitment of neutrophils and the subsequent bacterial clearance in *Legionella* pneumonia depend on IL-1R^47^, strengthens our hypothesis that the intercellular communication via cytokines and EVs is critical for the host immune response to bacterial infections.

Our study provides for the first time a detailed analysis of the differential effects of the released cytokines and EVs on different cell types in the context of *L*. *pneumophila* pneumonia. However, the influence of cytokines and EVs needs to be tested *in vivo*. This could help to further understand the pathogenesis of *Legionella*.

In summary, we report that intercellular communication in the context of *L*. *pneumophila* infection occurs via activation of surrounding alveolar epithelial cells by cytokines and exosomes. Bacterial OMVs activate resting bystander macrophages via TLR2. Culminating in an enhanced CXCL8 release, this might be beneficial to create a pro-inflammatory environment in order to mount an immune response, eventually clearing the infection by recruitment of neutrophils.

## Materials and Methods

### Chemicals and reagents

RPMI-1640 and FCS were obtained from Thermo Fisher Scientific Inc. (Waltham, USA). Ham’s F12 was obtained from GE Healthcare Europe (Freiburg, Germany). PBS was from Biochrom GmbH (Berlin, Germany). Phorbol 12-myristate 13-acetate (PMA) and GW4869 were purchased from Sigma-Aldrich Chemie GmbH (Taufkirchen, Germany). 4′,6-diamidino-2-phenylindole (DAPI) was from AAT Bioquest (Sunnyvale, USA). TLR2 blocking antibody was acquired from eBioscience (T2.5, San Diego, USA). Anti-LPS antibody (*L*. *pneumophila* specific; ABIN235748) was obtained from antikörper-online.de (Aachen, Germany). All other antibodies were purchased from Santa Cruz: anti-Alix (sc-49268), anti-Tsg101 (sc-7964), anti-CD63 (sc-5275), anti-CD81 (sc-7637), anti-GRP78 (sc-1050), anti-LaminA/C (sc-20681) and anti-tubulin (sc-5286). All chemicals used were of analytical grade and obtained from commercial sources.

### Bacterial strains and infection

*L*. *pneumophila* Corby wild type was kindly provided by A. Flieger, Wernigerode, Germany, and routinely grown as described^48^. Briefly, bacteria were plated on BCYE charcoal agar and incubated for 3 days at 37 °C and 5% CO_2_. For infection, bacteria were resuspended in PBS. Infection doses were calculated by OD_600_ measurement. THP-1 cells were infected for 24 h at multiplicity of infection (MOI) of 0.25 or 0.5.

### Cell culture

THP-1 and A549 cells were obtained from the American Type Culture Collection (ATCC) and cultured at 37 °C and 5% CO_2_ in RPMI-1640 or Ham’s F12 with 10% FCS without antibiotics, respectively. For all experiments, THP-1 cells were differentiated by addition of 20 nM PMA for 24 h. Murine bone marrow-derived macrophages (mBMDM) were prepared from femurs and tibiae of C57BL/6 N wild type and TLR2/4^−/−^ mice (kindly provided by M. Schnare, Marburg, Germany) and cultivated as described^49^. Briefly, bone marrow-derived cells were cultivated for 7 days in the consecutive presence of M-CSF and GM-CSF. Maturation was validated by microscopy.

### Quantitative RT-PCR

RNA isolation, DNase I digestion and quantitative RT-PCR were performed as described^22^. Briefly, RNA was isolated from THP-1 and A549 cells by phenol-chloroform precipitation. For pri-mir-146a measurement, purified RNA was DNase digested to eliminate contaminating traces of genomic DNA. For quantitative PCR (qPCR), the SYBR Green and Taqman probe detection methods were used. The following custom made primers were used for SYBR Green based qPCR: *RPS18*: fwd: 5′-GCGGCGGAAAATAGCCTTTG-3′, rev: 5′-GATCACACGTTCCACCTCATC-3′; *CXCL8*: fwd: 5′-ACTGAGAGTGATTGAGAGTGGAC-3′, rev: 5′-AACCCTCTGCACCCAGTTTTC-3′. *SOD2*: fwd: 5′-ATGTTGAGCCGGGCAGTGTG-3′, rev: 5′-GCGCGTTGATGTGAGGTTCC-3′; *TNFAIP2*: fwd: 5′-GACTTGGGCTCACAGATAAAGC-3′, rev: 5′-CAGGCAGTTGTTGATGTTGG-3′; *CXCL1*: fwd: 5′-AAGTCATAGCCACACTCAAGAATGG-3′, rev: 5′-TTCAGGAACAGCCACCAGTGAG-3′; *IL-1β*: fwd: 5′-AGCTCGCCAGTGAAATGATGG-3′, rev: 5′-CAGGTCCTGGAAGGAGCACTTC-3′; *IFN-β*: fwd: 5′-CCGCATTGACCATCTATGAG-3′, rev: 5′-AGACATTAGCCAGGAGGTTC-3′. Commercial microRNA and pri-microRNA Taqman primers were purchased from Thermo Fisher Scientific Inc. (RNU48: 001006, miR-146a-5p: 000468, pri-mir-16–2: hs03303046_pri, pri-mir-146a: hs03303259_pri). All samples were processed on a ViiA7 qPCR device (Life Technologies).

### Extracellular vesicle preparation and analysis

THP-1 cells were incubated in exosome-depleted medium and infected with *L*. *pneumophila* at indicated MOIs or left untreated for control. After 24 h, the supernatant was centrifuged at 1,000 × g for 15 min to remove dead cells. The supernatant was then centrifuged again at 16,000 × g for 30 min to pellet apoptotic bodies and microparticles, yielding the 16 K pellet. After sterile-filtration, the remaining supernatant was ultracentrifuged at 100,000 × g for 3 h to obtain the 100 K pellet, containing extracellular vesicles (EVs) such as exosomes and OMVs. Purified EVs were analysed by nanoparticle tracking analysis (NTA) in a NanoSight instrument (kindly provided by S. Dimmeler, Frankfurt, Germany). Transmission electron microscopic (TEM) analysis was performed to visualize vesicles and assess purity. After fixation of the 100 K sample with 0.5% glutaraldehyde and 2% formaldehyde for 15 min on ice and subsequent negative staining (2% uranyl acetate), samples were scanned using a JEOL 2100 TEM operated at 120 kV. Immunoprecipitation of CD63^+^ exosomes from the purified EV fraction was performed overnight on a rotator at 4 °C using the exosome-human CD63 isolation/detection reagent (Thermo Fisher Scientific Inc.). For an exosome-free EV fraction, THP-1 cells were incubated with the nSMase inhibitor GW4869 (10–15 µM) before infection and collection of supernatant after 24 h of infection.

### Stimulation experiments

Purified EVs from 2 × 10^6^ THP-1 cells were resuspended in RPMI and added to recipient cells (A549 or THP-1 cells). When indicated, recipient THP-1 cells were pre-incubated with TLR2 blocking antibody (α-TLR2, 20 µg/mL) for 90 min before administration of EVs. Cytokine-containing supernatant that was clear of EVs after 100,000 × g ultracentrifugation from control or infected THP-1 cells was added to the recipient cells at one sixth of the total volume. Recipient cells were incubated for 24 or 48 h, respectively. When indicated, TNF-α neutralizing antibody (α-TNF-α, 15 ng/mL) or IL-1 receptor antagonist (IL-1RA, 100 ng/mL; both R&D systems, Minneapolis, USA) were added 1 h before stimulation of recipient A549 cells with EV-free supernatant.

### Immunofluorescence

For immunofluorescence analysis of EV uptake, the membrane of EV-donor A549 cells was stained with the PKH67 membrane dye (Sigma-Aldrich Chemie GmbH) according to the manufacturer’s recommendations. The resulting dyed EV pellet was prepared as described above and incubated with recipient THP-1 cells for 1 or 3 h, respectively. Cells were fixed for 15 min with 4% paraformaldehyde and nuclei were stained by addition of DAPI. Pictures were taken on an Axio Vert.A1 Fluorescence Microscope with an AxioCam MRm (Zeiss, Oberkochen, Germany).

### ELISA

CXCL8 from the supernatant of THP-1 cells was analysed with the OptEIA ELISA kit (BD Biosciences, Heidelberg, Germany). CXCL1 from supernatant of mBMDM was analysed with the DuoSet ELISA kit (R&D systems). All other cytokines (IL-1β, IL-6, IL-10, MCP-1, GM-CSF, TNF-α) were measured in a Bio-Plex Magpix (Luminex Corporation, Austin, USA) with a custom made Luminex Assay (R&D systems) according to the manufacturer’s recommendations.

### Western Blot

Western blot was performed as described^50^. Briefly, samples were transferred by wet blot onto the membrane. After blocking and washing, immunodetection was carried out with the above-listed antibodies and visualized by HRP-mediated turnover of ECL substrate (GE Healthcare, Little Chalfont, UK) on a chemiluminescence imager (INTAS Science Imaging Instruments, Göttingen, Germany).

### Ethical Statement

Animals were handled according to the EU council directive 86/609/EEC for the protection of animals. The performed protocols were approved by the responsible animal ethics committee (Philipps-University Marburg; permit number: EX-22-2013).

### Statistical analyses

Data are presented as mean + SEM (standard error of the mean) of at least three independent experiments. Effects were statistically evaluated employing the non-parametric Mann-Whitney-U test or 2-way ANOVA. p-values < 0.05 were considered significant.

## Electronic supplementary material

Supplementary Information
