# Supplementary material for: Legionella pneumophila infection activates bystander cells differentially by bacterial and host cell vesicles
Source: Sci Rep. 2017 Jul 24;7:6301. doi: 10.1038/s41598-017-06443-1 (PMC5524687; doi:10.1038/s41598-017-06443-1)
Supplement: Supplementary file 1 — Supplementary Information [file 41598_2017_6443_MOESM1_ESM.pdf]

Legionella pneumophila infection activates bystander cells differentially by bacterial and host cell vesicles

Running Title: Extracellular vesicles in infection

Anna Lena Jung[1], Christina Elena Herkt[1], Christine Schulz[1], Kathrin Bolte[2], Kerstin Seidel[1], Nicoletta Scheller[1], Alexandra Sittka-Stark[1,3], Wilhelm Bertrams[1], Bernd Schmeck[1,4*]

1) Institute for Lung Research, German Center for Lung Research, Universities of Giessen and Marburg Lung Centre, Philipps-University Marburg, 35043 Marburg, Germany

2) Department for Cell Biology, Philipps-University Marburg, 35043 Marburg, Germany

3) Current address: Labor Berlin Services GmbH, 13353 Berlin, Germany

4) Department of Medicine, Pulmonary and Critical Care Medicine, University Medical Center Marburg, Philipps-University, 35043 Marburg, Germany

*) Corresponding author: Bernd Schmeck, phone: +49 (0)6421/ 28-21954; fax: +49 (0)6421/28-21993; Email: bernd.schmeck@uni-marburg.de.

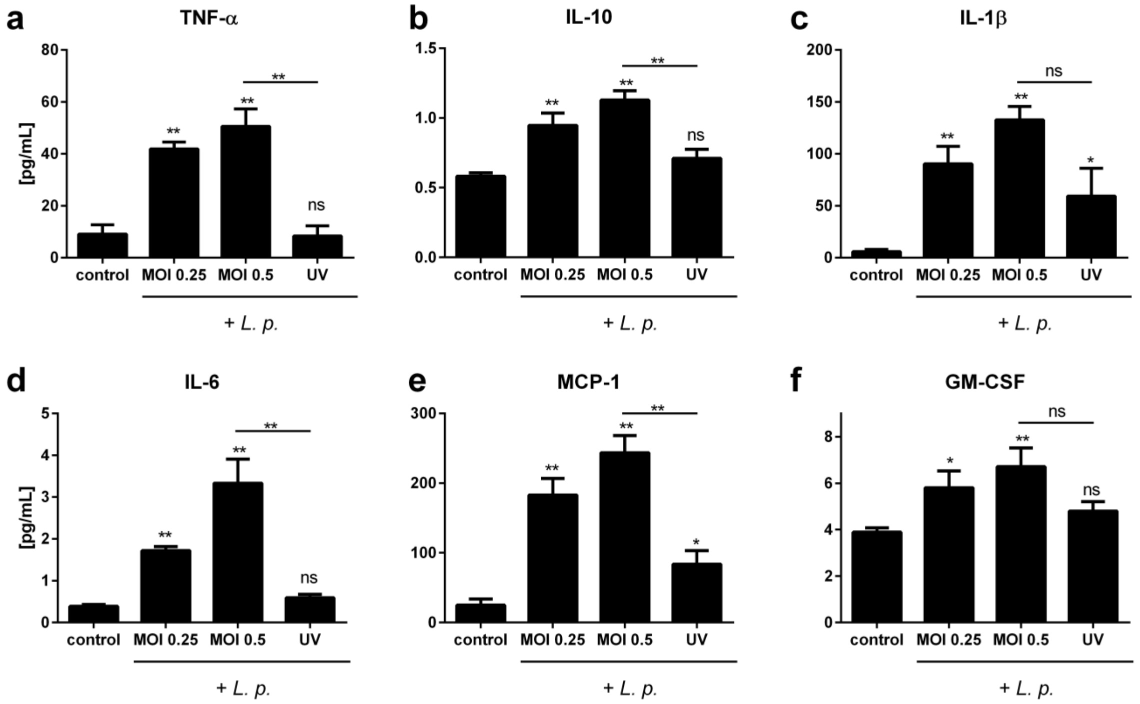

<u>Supplementary Figure 1:</u> Cytokine secretion of *L. pneumophila*-infected THP-1 cells. (a-f) THP-1 cells were infected with L. pneumophila (MOI 0.25 or 0.5), stimulated with UV-inactivated *Legionella* (MOI 0.5) or left untreated for control. Cytokine secretion profile of cell-free supernatant was assessed by multiplex ELISA assay. Results for TNF-α (a), IL-10 (b), IL-1β (c), IL-6 (d), MCP-1 (e) and GM-CSF (f) are presented. Data are shown as mean +SEM of three independent experiments. * $p<0.05$, ** $p<0.01$, ns: not significant.

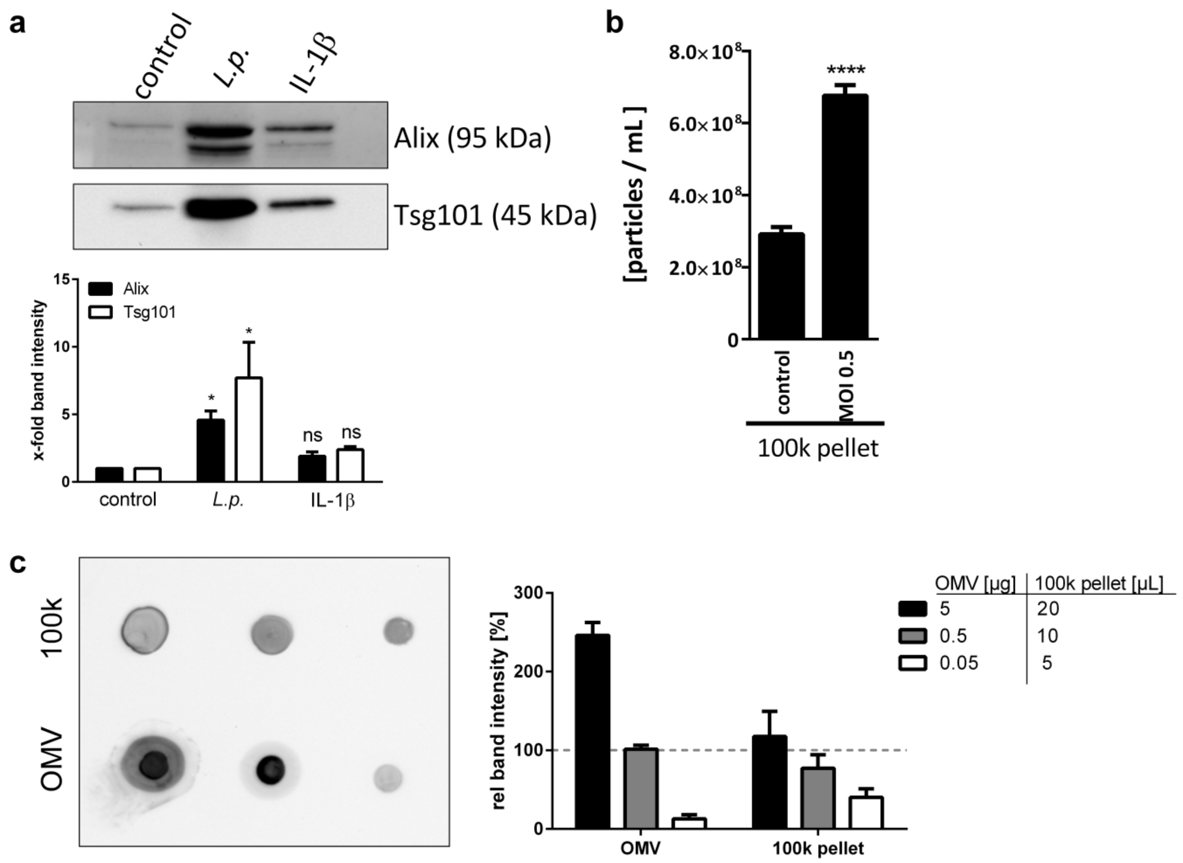

Supplementary Figure 2: ***L. pneumophila*** **infection increases the secretion of EVs in primary human macrophages.** (a) Increase in exosome release after *L. pneumophila* infection of THP-1 cells. THP-1 cells were infected with *L. pneumophila* MOI 0.5 for 24 h, stimulated with IL-1β (1 ng/mL) or left untreated for control. EVs were purified and the 100k pellet was used for western blot, which was stained for Alix and Tsg101. Quantification of three independent experiments is shown. (b) Amount of exosomes in response to *L. pneumophila* infection. NTA was performed with the 100k pellet prepared by differential centrifugation of human BDMs supernatant 24 h post infection with a MOI of 0.5. (c) 100k pellet contains *L. pneumophila* LPS. Dot blot was performed with indicated amounts of 100k pellet of *L. pneumophila*-infected THP-1 cells (MOI 0.5 for 24 h) and with purified OMVs as a reference (generated as previously described [17]). Data are shown as mean +SEM of three independent experiments. * $p<0.05$, ****$p<0.0001$, ns: not significant.

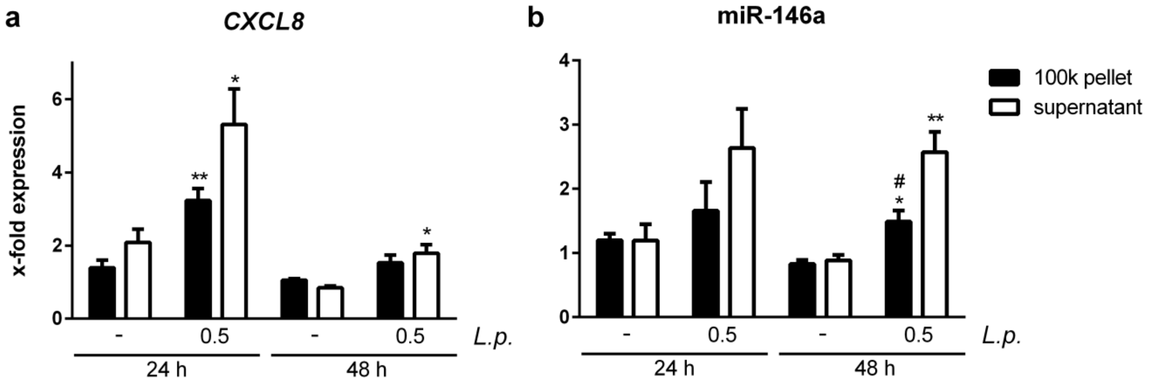

<u>Supplementary Figure 3:</u> Primary alveolar epithelial cells respond to EVs and cytokines. (A/B) Response of primary human alveolar epithelial cells to vesicle-free supernatant or 100k pellet from *L. pneumophila*-infected THP-1 cells. Primary human alveolar epithelial cells were stimulated with vesicle-free supernatant or 100k pellet from THP-1 cells infected with *L. pneumophila* (MOI 0.5 for 24 h) and incubated as indicated for 24 or 48 h, respectively. Expression of *IL-8* (A) and miR-146a (B) was analyzed by qPCR. Data are shown as mean +SEM of three independent experiments from different human donors. *p<0.05, **p<0.01. * compared to corresponding control, # compared to corresponding supernatant treated sample.

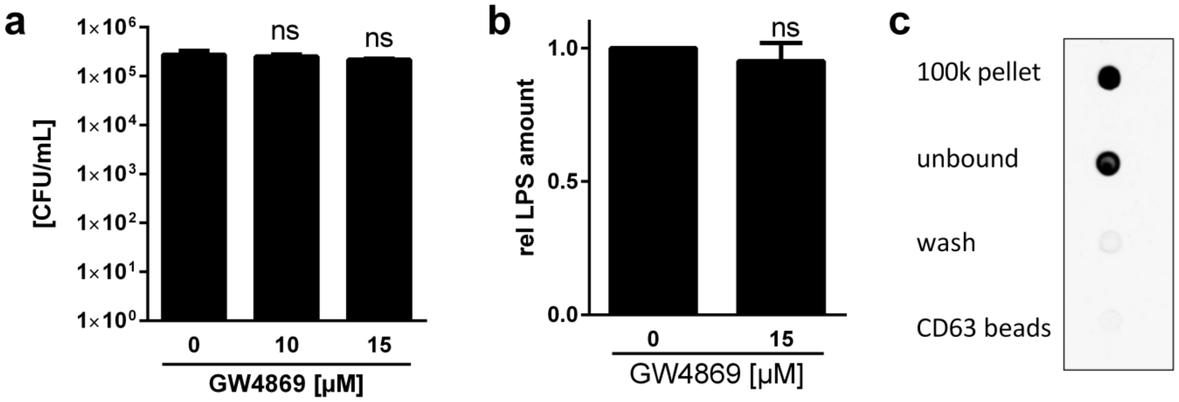

<u>Supplementary Figure 4:</u> GW4869 does not influence *L. pneumophila* replication and OMV secretion. (a) THP-1 cells were pre-incubated with GW4869 (10 or 15 µM) or DMSO for 1 h before infection with *L. pneumophila* (MOI 0.5) for 24 h. Bacterial replication was determined by colony forming unit (CFU) assay. Bars represent mean values +SEM from three biological independent experiments each performed in duplicates. (b) THP-1 cells were pre-incubated with 15 µM GW4869 or DMSO for 1 h before infection with *L. pneumophila* (MOI 0.5) for 24 h. 100k pellet was generated and used for dot blot with LPS antibody. The relative dot intensity of three independent experiments is shown. (c) LPS dot blot after CD63 immunoprecipitation of 100k pellet. Entire 100k pellet, unbound fraction, washing step and CD63 beads were loaded. Data are shown as mean +SEM of three independent experiments. ns: not significant.

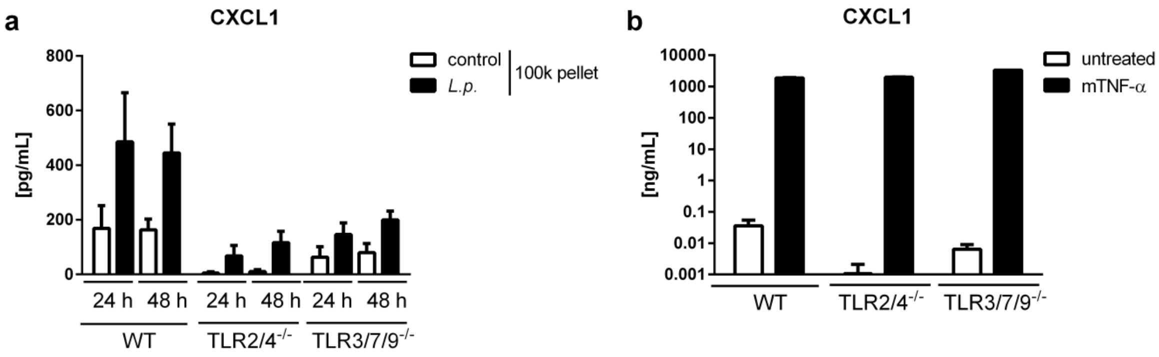

<u>**Supplementary Figure 5:**</u> **EVs signal via TLR 2/4 and TLR3/7/9 in macrophages.** (a) Response of mBMDM to 100k pellet from *L. pneumophila*-infected THP-1 cells. mBMDM were stimulated with EVs from THP-1 cells infected with *L. pneumophila* (MOI 0.5 for 24 h) for 24 and 48 h, respectively. Supernatant was collected for CXCL1-ELISA. Data are shown as mean +SEM of three independent experiments. 2-way ANOVA: treatment: **** ($p<0.0001$); genotype: **** ($p<0.0001$); interaction: * ($p<0.0476$). (b) CXCL1 release of mBMDM after mTNF-α treatment (400 ng/mL). Data are shown as mean +SEM of three independent experiments.

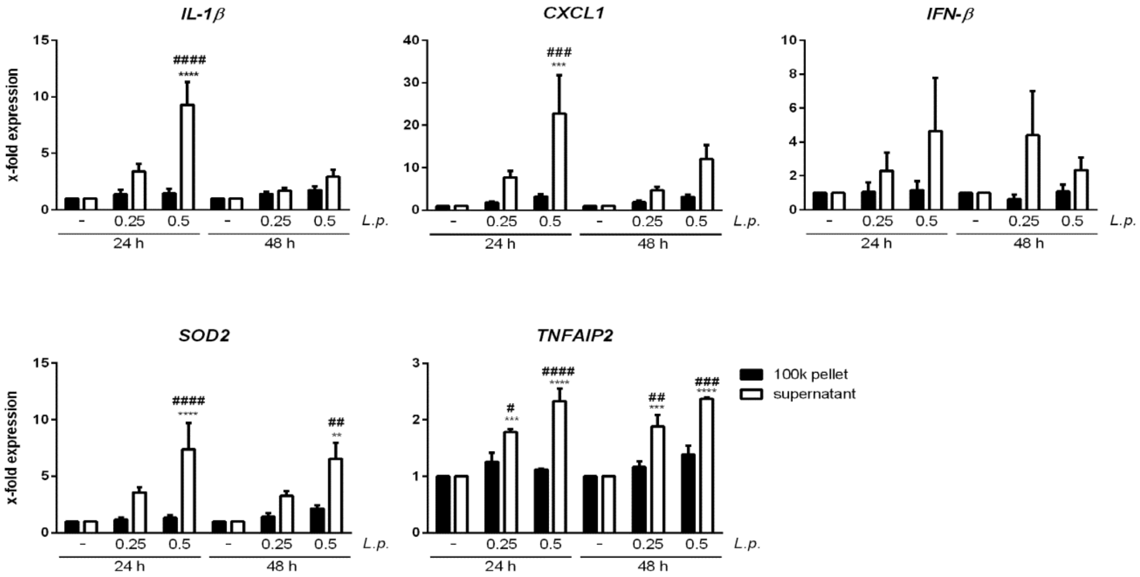

<u>Supplementary Figure 6:</u> *L. pneumophila* induced cytokines elicit a pro-inflammatory response in non-infected A549 cells. Response of A549 cells to vesicle-free supernatant or 100k pellet from *L. pneumophila*-infected THP-1 cells. A549 cells were stimulated with vesicle-free supernatant or 100k pellet from THP-1 cells infected with *L. pneumophila* (*L.p.*) at an MOI of 0.25 and 0.5, respectively, for 24 h. A549 cells were incubated as indicated for 24 or 48 h, respectively. RNA and supernatant were collected. qPCR was performed for expression of *SOD2*, *CXCL1*, *IFN-b*, *IL-1b* and *TNFAIP2*. Data are shown as mean +SEM of at least three independent experiments. *p<0.05, **p<0.01, ***p<0.001, ****p<0.0001. * compared to corresponding control, # compared to corresponding supernatant treated sample.

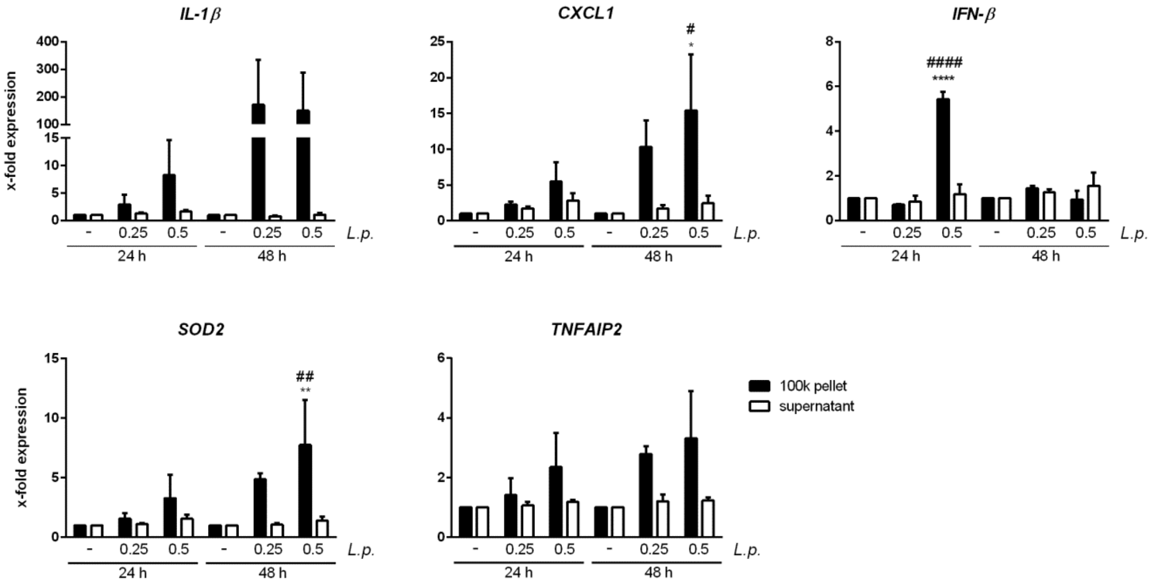

<u>Supplementary Figure 7:</u> *L. pneumophila* induced EVs elicit a pro-inflammatory response in non-infected THP-1 cells. Response of THP-1 cells to vesicle-free supernatant or 100k pellet from *L. pneumophila*-infected THP-1 cells. THP-1 cells were stimulated with vesicle-free supernatant or 100k pellet from THP-1 cells infected with *L. pneumophila* (MOI 0.25 and 0.5, respectively) for 24 h. Recipient THP-1 cells were incubated for 24 or 48 h, respectively. qPCR was performed for expression of *SOD2*, *CXCL1*, *IFN-b*, *IL-1b* and *TNFAIP2*. Data are shown as mean +SEM of at least three independent experiments. *p<0.05, **p<0.01, ***p<0.001, ****p<0.0001. * compared to corresponding control, # compared to corresponding supernatant treated sample.

Activation Marker Gene (Symbol, Family, Entrez Gene ID)	Associated Diseases and Functions, p-value
CCL2, cytokine, 6347	attraction of leukocytes, 1.76E-12
CXCL1, cytokine, 2919	
CXCL8, cytokine, 3576	
IFN Beta, group, #	apoptosis of leukocytes, 4.16E-09
SOD2, enzyme, 6648	
TNFAIP2, other, 7127	
IL1B, cytokine, 3553	organization of cytoskeleton, 2.15E-06

<u>Supplementary Figure 8</u>**: Induced markers in A549 and THP-1 recipient cells indicate broad pro-inflammatory activation.** Qiagen Ingenuity Pathway Analysis of the measured markers (grey shaded area) yields associated diseases and functions that illustrate the mounting of an immune response upon treatment with supernatant or 100K pellet.
